# Comparison of Seven Non-Linear Mixed Effect Model-Based Approaches to Test for Treatment Effect

**DOI:** 10.3390/pharmaceutics15020460

**Published:** 2023-01-30

**Authors:** Estelle Chasseloup, Mats O. Karlsson

**Affiliations:** Pharmacometrics Group, Pharmacy Department, Uppsala University, 751 23 Uppsala, Sweden

**Keywords:** individual model averaging, model averaging, randomization test, Likelihood Ratio Test, longitudinal modelling, type I error, power, accuracy, model misspecification

## Abstract

Analyses of longitudinal data with non-linear mixed-effects models (NLMEM) are typically associated with high power, but sometimes at the cost of inflated type I error. Approaches to overcome this problem were published recently, such as model-averaging across drug models (MAD), individual model-averaging (IMA), and combined Likelihood Ratio Test (cLRT). This work aimed to assess seven NLMEM approaches in the same framework: treatment effect assessment in balanced two-armed designs using real natural history data with or without the addition of simulated treatment effect. The approaches are MAD, IMA, cLRT, standard model selection (STDs), structural similarity selection (SSs), randomized cLRT (rcLRT), and model-averaging across placebo and drug models (MAPD). The assessment included type I error, using Alzheimer’s Disease Assessment Scale-cognitive (ADAS-cog) scores from 817 untreated patients and power and accuracy in the treatment effect estimates after the addition of simulated treatment effects. The model selection and averaging among a set of pre-selected candidate models were driven by the Akaike information criteria (AIC). The type I error rate was controlled only for IMA and rcLRT; the inflation observed otherwise was explained by the placebo model misspecification and selection bias. Both IMA and rcLRT had reasonable power and accuracy except under a low typical treatment effect.

## 1. Introduction

Population model-based (pharmacometric) approaches, through the usage of NLMEM, improve the power considerably when analyzing longitudinal data [1,2,3,4]. However, the assumptions involved in NLMEM, e.g., the absence of model misspecification or asymptotic conditions, can impact the performance of such approaches in terms of type I error, power, and accuracy of the treatment effect estimates [5]. As the development of a reasonable model often implies a data-driven trial and error process across many models, type I error inflation related to multiple testing is a legitimate concern. Furthermore, despite all the efforts invested in the rationalization of the selection of one of the candidate models, it inevitably leads to selection bias, and relying on a unique selected model can hinder inference by discarding the model structure uncertainty and dismissing the inherent model misspecification [6].

Over the recent years, multiple approaches have been developed to overcome these caveats. Model-averaging across drug models (MAD) weights the outcome of interest from a set of pre-selected models according to a goodness-of-fit based metric [7,8,9,10] to prevent selection bias and handle model structure uncertainty. Individual model averaging (IMA) [11] uses mixture models to test for treatment effect, which mitigates consequences of both placebo and drug model misspecification and improves the conditions of application of the likelihood ratio test (LRT). combined-LRT (cLRT) [12] combines an alternative cut-off value for the LRT and MAD to handle model structure uncertainty.

The pre-selection of a set of possible candidate models prior to the data analysis, recommended in the ICH E9 guidance [13], is a common alternative to handle model selection bias and its consequences in terms of bias in the estimates. The restriction of the set of candidate models also inherently reduces the type I error inflation caused by multiple testing. MAD, IMA, and cLRT were assessed separately in different contexts of treatment effect or dose-response assessment using real or simulated data. This work aimed to assess MAD, IMA, and cLRT together with four other related approaches in the same framework: treatment effect assessment in balanced two-armed designs using real data. The additional approaches were standard model selection (STDs), structural similarity selection (SSs), randomized-cLRT (rcLRT), and model-averaging across placebo and drug models (MAPD).

Three evaluation aspects were considered: type I error, power, and accuracy of treatment effect estimate (assessed via the root mean squared error (RMSE)). The former aspect was assessed using real natural history data, while the two latter were assessed on the same natural history data modified by the addition of various simulated treatment effects. Model candidate pre-selection is an inherent part of the model-averaging approaches. In this work, it was generalized to all the approaches to provide a common scope to the seven NLMEM approaches for the evaluation. The AIC was used for selection and weighting according to previous recommendations [8,9].

## 2. Materials and Methods

For parameter estimation, NONMEM [14] version 7.5.0 was used. The simulation or randomization and re-estimations were performed using PsN [15,16] version 5.2.1 through the Stochastic Simulation and Estimation or the randtest functions. The runs with failed minimization status or unreportable number of significant digits were removed from the analysis (see Appendix A for more details). The first order with conditional estimates (FOCE) method was used for all models without the interaction option, as the residual error model was additive. The processing of the results was performed with the statistical software R [17] version 4.1.2.

### 2.1. Data

Data used in the preparation of this article were obtained from the Alzheimer’s Disease Neuroimaging Initiative (ADNI) database (adni.loni.usc.edu). The ADNI was launched in 2003 as a public-private partnership, led by Principal Investigator Michael W. Weiner, MD. The primary goal of ADNI has been to test whether serial magnetic resonance imaging, positron emission tomography, other biological markers, and clinical and neuropsychological assessment can be combined to measure the progression of mild cognitive impairment and early Alzheimer’s disease. For up-to-date information, see www.adni-info.org.

The real natural history data were longitudinal ADAS-cog scores ranging from 0 to 70, previously published and detailed elsewhere [18]. Due to the high number of categories, the data were treated as continuous. In this work, we used 817 individuals (aged from 55 to 91 years old), with ADAS-cog evaluation at 0, 6, 12, 18, 24, and 36 months, for a total observation count of 3597. The Baseline Mini-Mental State (BMMS) was also collected at baseline for all the individuals and is used to describe the baseline ADAS-cog scores.

The study population was randomized to two study arms, representing placebo (TRT=0) and treatment (TRT=1). In the base scenario used to assess type I error, all subjects’ data were their natural disease progression. To assess the power and the accuracy of the treatment effect estimates, the original data were also modified by adding various treatment effect functions to the individual allocated to the treated arm. Offset (Equation (2)) and time-linear (Equation (3)) models were used to generate different treatment effect scenarios: with (30% CV) or without IIV on the treatment effect parameters, with a low (2-points increase) or a high (8-points increase) typical treatment effect at the end of the study. Eight treatment effect scenarios were generated, using both time-linear and offset drug models: (1) with or; (2) without IIV; (3) small treatment effect; and (4) large treatment effect.

### 2.2. Models

The published disease model is described extensively elsewhere [18] and summarized in Equation (1). The corresponding NONMEM code is provided in Appendix B. The disease model is time-linear (Equation (1a)), including covariates effects on the slope (Equation (1c)), and a slope model links the baseline value to BMMS (Equation (1b)).

(1a) $$\begin{array}{cc} {\mathrm{ADAS}}_{\mathrm{cog},i}\left(t\right) & ={\mathrm{ADAS}}_{\mathrm{cog},i}\left(0\right)+\alpha_{i}t+\varepsilon \end{array}$$

(1b) $$\begin{array}{cc} {\mathrm{ADAS}}_{\mathrm{cog},i}\left(0\right) & =\left(\Theta_{\mathrm{baseline}}+\Theta_{\mathrm{intercept}}\cdot {\mathrm{BMMS}}_{i}\right)+\eta_{1,i} \end{array}$$

(1c) $$\begin{array}{cc} \alpha_{i} & =f({\mathrm{Cov}}_{i},\Theta ,\eta_{2,i}) \end{array}$$

where Θ describes fixed effect parameters, $\eta_{i}∼N\left(0,\omega^{2}\right)$ are additive individual random effects, and $\varepsilon ∼N(0,\sigma^{2})$ is the residual error for each observation. Four alternative disease models were considered for MAPD and are presented in Table 1.

Offset with or without IIV (Equation (2)) and disease-modifying with or without IIV (Equation (4)) models were considered as treatment effect models for the type I error assessment. For the power and accuracy assessment, a time-linear model (Equation (3)) was used instead of the disease-modifying model to avoid any disease model assumption in the simulation of the treatment effect.

(2) $$\Theta_{\mathrm{DE}}+\eta$$

(3) $$\frac{\Theta_{\mathrm{DE}}+\eta }{36}t$$

(4) $$\alpha (1-\left(\Theta_{\mathrm{DE}}+\eta \right))$$

With α being the disease model slope.

### 2.3. Description of Modelling Approaches

For all the approaches, the Akaike information criteria (AIC) is used to compare the fit of the set of candidate models. The AIC is hence used to select the best-fitting candidate used as the alternative hypothesis (H1) in the statistical test, except for the model-averaging approaches, i.e., MAD and MAPD, for which no selection occurs, but an AIC-based weight is computed for each candidate model. The LRT is then used to conclude the presence of a treatment effect, except for the model averaging approaches, cLRT, and rcLRT for which the alternatives are described below.

**Figure 1:**
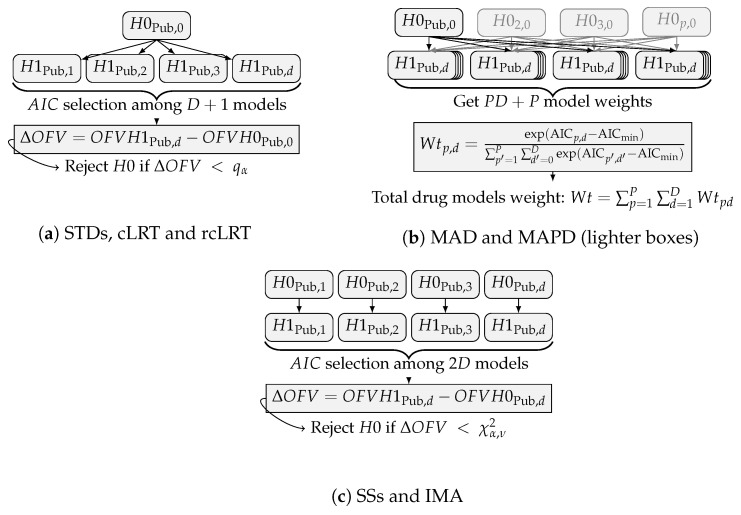
Workflow illustration of the different methods.

In the STDs approach (Equation (5), Figure 1a), the null hypothesis (H0) consists of a placebo model applied to all subjects, and H1 adds a drug model to the treated subjects. The LRT is used to discriminate between the best model selected and H0 to conclude the presence of a treatment effect, using ΔOFV as the test statistic. The distribution of this test statistic under H0 is unknown. In the LRT, it is assumed to follow a $\chi^{2}$ distribution with ν degrees of freedom, with α=0.05, and ν, the number of additional parameters estimated in H1 compared to H0. cLRT and rcLRT assumed a different distribution for that test statistic under H0, the alternative distribution being obtained by replicating the model selection procedure and the test statistic computation n=100 times over *n* different data sets. For cLRT (Equation (5), Figure 1a), the distribution is obtained with *n* data sets simulated under H0, for rcLRT (Equation (5), Figure 1a) the distribution is obtained with *n* randomized data set differing by the treatment allocation assignment.

In SSs (Equation (6), Figure 1c), the drug model is fitted to all subjects in H0, but H1 allows different estimates for the treated individuals. The LRT is used to conclude on the presence of a treatment effect using the best model according to the AIC.

The two model averaging approaches, MAD and MAPD, have the H0 and H1 hypotheses constructed according to STDs (Equation (5) and Figure 1b). Instead of selecting a unique best candidate model via a selection step, the model-averaging approaches assigned an AIC-based weight to each candidate model (Equation (7)), with ${\mathrm{AIC}}_{\min}$ being the minimum AIC of the candidate models. Hence, each model from the pre-defined set contributes to the computation of the metric of interest proportionally to its relative weight, contrary to the selection-based methods where only the best model candidate is used to draw conclusions. MAD considered a unique H0 and multiple H1 via the formulation of various drug models and a unique placebo model, while MAPD differed by also considering various placebo models in the set of pre-defined models. In that aspect, MAPD differed from MAD, and the other six approaches, by considering multiple disease models instead of only the published disease model.

In IMA (Equation (8a), Figure 1c), all subjects have, through a mixture feature, a probability $\Theta_{\mathrm{MIX}}$ of being described by the drug model. This probability is fixed to the placebo allocation rate (0.5) in H0 but estimated based on the treatment allocation in H1. The LRT is used to conclude on the presence of a treatment effect using the best model according to the AIC.

(5a) $$\begin{array}{cc} H0_{\mathrm{Pub},0} & :{\mathrm{Plb}}_{\mathrm{Pub}} \end{array}$$

(5b) $$\begin{array}{cc} H1_{\mathrm{Pub},d} & :{\mathrm{Plb}}_{\mathrm{Pub}}+f_{\mathrm{drug},d}\left(TRT\right) \end{array}$$

where ${\mathrm{Plb}}_{\mathrm{Pub}}$ is the published placebo model and $f_{\mathrm{drug},d}\left(TRT\right)$ a drug model *d* depending on the treatment allocation TRT.

(6a) $$\begin{array}{cc} H0_{\mathrm{Pub},d} & :{\mathrm{Plb}}_{\mathrm{Pub}}+f_{\mathrm{drug},d} \end{array}$$

(6b) $$\begin{array}{cc} H1_{\mathrm{Pub},d} & :{\mathrm{Plb}}_{\mathrm{Pub}}+\left\{\begin{array}{cc} f_{\mathrm{drug},d} & \mathrm{if}\;TRT=0,\\ f_{\mathrm{drug},d} & \mathrm{if}\;TRT=1, \end{array}\right. \end{array}$$

where the same drug model *d* is applied to all the individuals, allowing for different parameter estimates between the two arms in H1.

(7) $$\begin{array}{c} Wt_{p,d}=\frac{\exp({\mathrm{AIC}}_{p,d}-{\mathrm{AIC}}_{\min})}{\sum_{p^{\prime }=1}^{P}\sum_{d^{\prime }=0}^{D}\exp\left({\mathrm{AIC}}_{p^{\prime },d^{\prime }}-{\mathrm{AIC}}_{\min}\right)} \end{array}$$

(8a) $$\begin{array}{c} \mathrm{Mixture}\mathrm{model}:\left\{\begin{array}{cc} {\mathrm{Plb}}_{\mathrm{Pub}} & \mathrm{if}\;\mathrm{Mix}=1\\ {\mathrm{Plb}}_{\mathrm{Pub}}+f_{\mathrm{drug},d} & \mathrm{if}\;\mathrm{Mix}=2 \end{array}\right. \end{array}$$

(8b) $$\begin{array}{cc} H0_{\mathrm{Pub},d}: & \;\;\;Pr\left(Mix=1\right)=Pr\left(Mix=2\right)=\Theta_{\mathrm{MIX}}=0.5\;\mathrm{FIX} \end{array}$$

(8c) $$\begin{array}{cc} H1_{\mathrm{Pub},d}: & \left\{\begin{array}{c} Pr\left(Mix=1\right)=\left(1-\mathrm{TRT}\right)\Theta_{\mathrm{MIX}}+\mathrm{TRT}\left(1-\Theta_{\mathrm{MIX}}\right)\\ Pr(Mix=2)=1-Pr(Mix=1) \end{array}\right. \end{array}$$

### 2.4. Approaches Assessment

For each of the seven approaches, the type I error rate was assessed first using the raw natural history data modified to randomly allocate (1:1) each subject to an artificial placebo or treated arm. The allocation was repeated N=100 times to mimic *N* random trials without treatment effect. The type I error rate was computed over the *N* trials as the frequency with which H0 was rejected and assumed to be adequate when falling within the 2.5th–97.5th percentiles of a binomial distribution with a probability of success of 5% on *N* trial replicates.

When the type I error was controlled, power and accuracy were assessed using the data modified by the addition of a treatment effect to the subjects allocated to the treated arm. *N* simulations were performed for each of the eight treatment effect scenarios. The power was computed as the frequency with which H0 was rejected over *N* trials. Regarding the model-averaging approaches, the type I error and power were computed as the percentage of the weights allocated to any of the H1 considered in the set of the candidate models.

The accuracy in the treatment effect estimates was assessed only when using the data modified by the addition of simulated treatment effect, using the RMSE according to Equation (9), where $\Theta_{\mathrm{DE},i}$ is the true value used in the simulations and ${\hat{\Theta }}_{\mathrm{DE},i}$ is the estimated value of the $n^{\mathrm{th}}$ trial.

(9) $$\mathrm{RMSE}=\sqrt{\frac{\sum_{n=i}^{N}{\left({\hat{\Theta }}_{\mathrm{DE},i}-\Theta_{\mathrm{DE},i}\right)}^{2}}{100}}$$

For IMA, ${\hat{\Theta }}_{\mathrm{DE},i}$ was computed according to Equation (10), to account for the submodel allocation probability:

(10) $${\hat{\Theta }}_{\mathrm{DE},i,\mathrm{IMA}}=\left(2\Theta_{\mathrm{MIX},i}-1\right){\hat{\Theta }}_{\mathrm{DE},i}$$

## 3. Results

**Figure 2:**
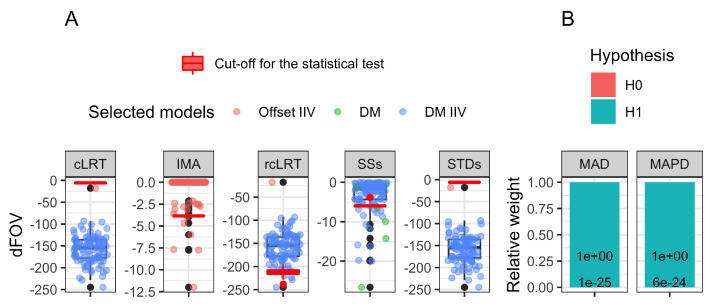
Panel (**A**) illustrates the type I error results for the non-model-averaging approaches: the colored dots and the associated black boxplot correspond to the distribution of the dOFV of the H1 hypothesis selected by the AIC selection step in each of the 100 trials. The distribution of the critical value used for the statistical test for each approach is indicated by the red boxplot. Panel (**B**) illustrates the proportion of the total relative weight associated with either the H0 or the H1 hypothesis.

**Figure A1:**
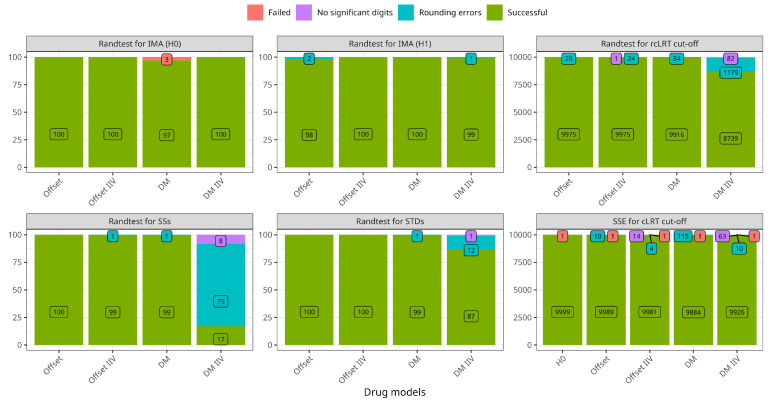
Minimization status for the models fitted on the natural history data for all the approaches but MAPD. Numbers indicate the count per status. DM: disease-modifying, IIV: inter-individual variability, SSE: stochastic simulation and estimation.

**Figure A2:**
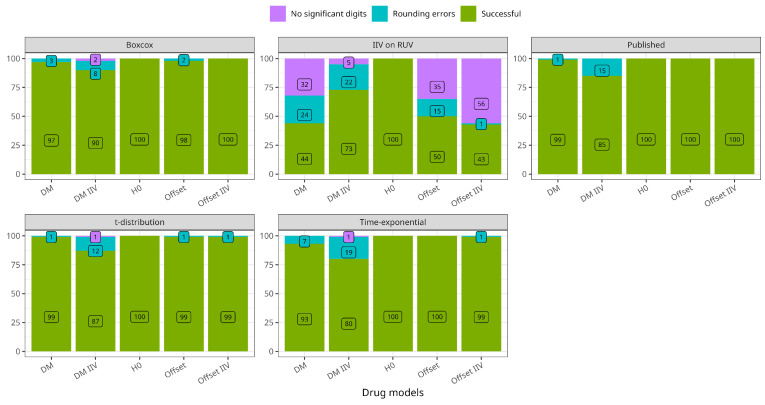
Minimization status for the MAPD approach on natural history data, facetted by placebo models. Numbers indicate the count per status. DM: disease-modifying, IIV: inter-individual variability, RUV: residual unexplained variability.

The type I error for each approach is available in Table 2. Only IMA and rcLRT had controlled type I error (6%). All the other approaches had 100% type I error except SSs, for which the type I error was inflated to 17%. The model-averaging approaches had a very negligible total weight assigned to any H0 hypothesis, $\leq 6\times 10^{-24}$. Details about the drug models selected in the *N* trials, their corresponding dOFV, and critical cut-off value for the LRT are presented in Figure 2A for all but the model-averaging approaches. The model-averaging approaches results are presented in Figure 2B, with the total relative weight allocated to any of the H0 or the H1 hypotheses. The minimization status is available in Appendix A in Figure A1 and Figure A2. The cLRT and rcLRT alternative distributions used for the determination of the cut-off value in the statistical test are presented in Figure A5 in Appendix C. The summary of the model fits (number of estimated parameters and OFV) is provided in Appendix D in Table A1 for the models used in the type I error computation for all the approaches but MAPD, and in Table A2 for the models used in MAPD, showing that the four proposed alternative disease models for MAPD improved the OFV significantly compared to the published disease model.

**Figure A3:**
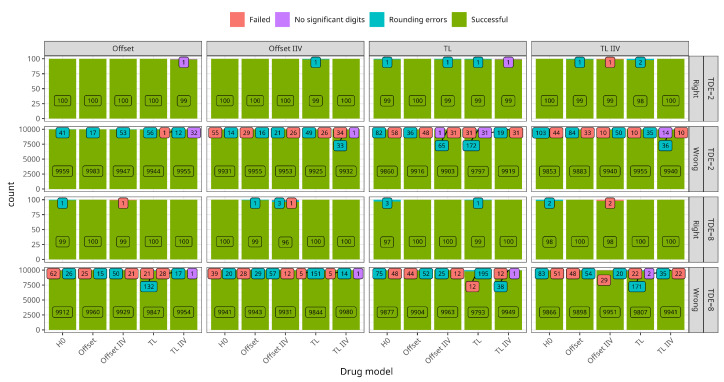
Minimization status for the rcLRT approach on data with various addition of simulated treatment effects. The plot is facetted horizontally by the function used to simulate the treatment effect and vertically by the typical size of the treatment effect and the treatment allocation used. Numbers indicate the count per status. IIV: inter-individual variability, TDE: typical drug effect, TL: time-linear.

**Figure A4:**
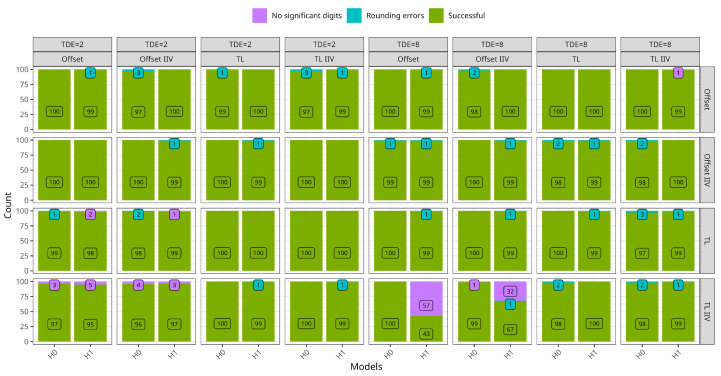
Minimization status for the IMA approach on data with various addition of simulated treatment effects. The plot is facetted horizontally by the function used to simulate the treatment effect and its typical size and vertically by the drug model fitted. Numbers indicate the count per status. IIV: inter-individual variability, TDE: typical drug effect, TL: time-linear.

**Figure A5:**
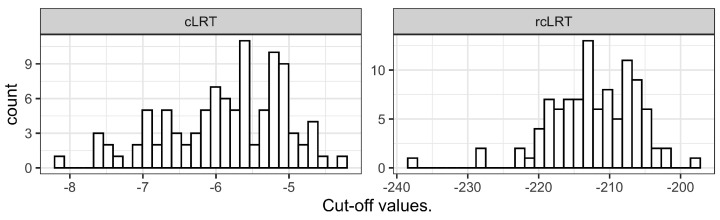
Distribution on the N cut-off values computed from simulations of H0 for cLRT (**left panel**), or from randomizations of the treatment allocation column for rcLRT (**right panel**). Each of the N cut-offs was taken as the 5th percentile of dOFV (H1-H0) computed over n = 100 data sets.

Power and accuracy in treatment effect estimates (RMSE) were investigated for IMA and rcLRT as they were the only two approaches with controlled type I error. The results (power and RMSE) for the eight investigated treatment effect scenarios are presented in Table 3. The minimization status is available in Appendix A.2 in Figure A3 for rcLRT, and in Figure A4 for IMA. For the high typical treatment effect scenarios (8-points), IMA and rcLRT had 100% power regardless of the simulated treatment effect model addition. For the low typical treatment effect scenarios (2-points), rcLRT had higher power than IMA when simulating the treatment effect with the offset models, whereas the opposite was true when simulating with the time-linear model. The RMSE was always higher for IMA for all eight scenarios tested.

## 4. Discussion

Seven NLMEM approaches were compared in the same context of treatment effect assessment in balanced two-armed trials using real natural history data. The comparison scope was first the type I error using the natural history data observed without any treatment. For approaches with controlled type I error, power and accuracy in the drug estimates were evaluated using the natural history data modified by the addition of different simulated treatment effects. Among the seven approaches tested, only two (IMA and rcLRT) had controlled type I error and were consequently assessed on data with a simulated treatment effect. IMA and rcLRT had similar results in terms of power: 100% power in the presence of a high typical treatment effect but lower in the presence of a low typical treatment effect, except for rcLRT when an offset drug model was used to simulate the treatment effect (100% power). rcLRT had consistently better RMSE than IMA.

The STDs approach type I error results (100%) could be anticipated from the fit of the four drug models on one randomization of the treatment allocation (see Table A1 in Appendix D.1). Out of the four models, offset or disease-modifying with or without IIV, the two models with IIV had a significant drop in OFV, according to the LRT. The disease-modifying model with IIV had a drop of −133.54, compared to a critical value of −5.99 for the LRT, about 111 OFV points lower than the offset drug model with IIV, leaving no chance of selection for another candidate model even after the parameters-based penalty introduced by the AIC. Previous investigations [11] of the STDs approach without the AIC selection step already outlined the uncontrolled type I error of the approach. Such uncontrolled type I error was attributed to the placebo model misspecification leaving room for additional model components and other possible violations of the standard LRT assumptions, such as not fulfilling the asymptotic properties. In this case, there was a pre-selection of H1 models using AIC. Another common way of model selection is to make multiple tests of different H1s against the H0 and then select the H1 associated with the lowest *p*-value, given that it is below the predetermined cut-off. Both these procedures suffer from multiple testing and their greedy behavior.

The cLRT approach [12] was introduced to account for the multiple testing of drug models and the structure model uncertainty in the computation of the critical value by using Monte Carlo simulation under H0. cLRT had controlled type I error in the context of simulated data [12], but had a 100% type I error inflation with the real natural history data and the published disease model that was used in our study. The alternative computation of cut-off values for cLRT was unable to prevent the type I error inflation.

Even though cLRT accounts for multiple testing in the computation of the critical value via Monte Carlo simulations, it still assumes that the structure of the placebo model is adequate by simulating under the assumption of that model for the computation of an alternative cut-off value for the statistical test. By computing the critical value using randomization of the treatment allocation, rcLRT adds the uncertainty of the placebo model in the computation of the critical value by removing any placebo model assumption from the process. The success of this approach (controlled type I error with a rate of 6%) could also be anticipated from the fit of the drug model on the natural history data (see Table A1 in Appendix D.1), as the dOFV of the best drug model used to compute the critical value is the same as the one used to test for treatment effect. This ensures that the distribution used for the critical value computation is of the same magnitude as the model selected by the AIC step, which is critical to have a chance to limit the type I error inflation. Appendix C illustrates the consequent difference in the typical value of the cut-off distribution obtained by cLRT and rcLRT, ranging, respectively, between −2 to −8 and −195 to −240. The success of this approach also validates the assumption that placebo model misspecification is the major factor involved in the type I error inflation of STDs and cLRT.

Aside from alternatives to the cut-off value used in the statistical test, SSs proposes another alternative to control the type I error inflation observed with STDs. SSs challenged the assumption of the main inflation factor being that the drug model tested is describing some features of the data that were not included in H0. Accordingly, SSs fits the drug model to all the subjects in H0 and allows for different estimates between the arms in H1. The expectation was that the drop in OFV observed in H1 for STDs, corresponding to an improvement of the placebo model rather than a treatment effect, would be included in the OFV of H0 and hence removed from the dOFV between H1 and H0. The results showed that the approach helped to decrease the type I error inflation (17% instead of 100%) but was not enough to control it. Further investigations would be necessary to decide whether and to which extent the remaining inflation should be attributed to multiple testing or the magnitude of the placebo model misspecification still present.

Pre-selection of the set of candidate drug models prior to the data analysis is a recommended practice to limit the type I error inflation [13]. Previous publications showed its application with NLMEM in combination with model-averaging techniques, which was helpful to integrate drug model misspecification in the prediction of key metrics to plan better later stages of drug development [8,9,12]. To our knowledge, in the NLMEM context, the averaging step was in these studies performed over a set of multiple drug model candidates and not over a set of both placebo and drug model candidates. In this work, the MAD approach illustrates the former, and MAPD the latter. MAD showed type I error control in previous publications on simulated data (method 3 from [8]) which was not the case with the real natural history data used in our study (type I error rate of 100%). For the model-averaging approaches, the type I error was computed as the percentage of the relative weights assigned to any H1, as the weights are usually used to favor the output of the respective models in the computation of an effect metric. Because the weights were AIC based, the favored models among the set of candidates were also the model with the lowest OFV (disease-modifying model with IIV), and because of the significant gap between this lowest and the second lowest OFV model (111 points), the total relative weight assigned to any H0 was very negligible ($10^{-25}$). This result was also predictable from the model fit on a single allocation randomization (see Table A1 in Appendix D.1). The addition of multiple placebo models in the set of candidate models did not help to reduce the type I error inflation and also resulted in a 100% type I error rate, even though the four alternative placebo models proposed all significantly improved the OFV (between −23.62 and −60.15 decrease in OFV). For the Boxcox transformation, the t-distribution, and the time-exponential model, the dOFV pattern across the four drug models was the same as with the published drug model. The drug models with IIV had significant dOFV, with the disease-modifying model with IIV being the best one, with a dOFV of about −100 points. The model with IIV on RUV had only the disease-modifying model with IIV as a significant treatment effect model with a drop of −68.84 points. The maximum difference between the model with the lowest OFV (13,585.26 for the t-distribution placebo model with disease-modifying with IIV model) and the model with the highest OFV (13,768.66 for the published model without treatment effect), i.e., 183.40 points, also lead to a very negligible total relative weight ($6\times 10^{-24}$) assigned to any H0. We can note that the multiplicity of H0 increased the total weight assigned to the H0 by less than $10^{-25}$. Both MAD and MAPD suffered from the gap in OFV between the published model without treatment effect and the model with the best treatment effect, even though the set of pre-selected drug model candidates is restricted to only four models.

Power and bias in treatment effect estimates were assessed for IMA and rcLRT on the natural history data modified by the addition of offset or disease-modifying treatment effects with or without IIV, with a low or a high typical treatment effect. Both approaches had similar power performances and reasonably good RMSE in the presence of a high typical treatment effect. However, in the presence of a low typical treatment effect simulated with an offset model, only rcLRT had good power and RMSE. When using time-linear treatment effect models with a low typical treatment effect, both IMA and rcLRT had unsatisfactory power and poor RMSE. These poor performances can be explained by the combination of two main factors: (1) a difficulty to distinguish the drug model from the placebo model as the added treatment effect was simulated with the same mathematical function as the placebo model; (2) the magnitude of the treatment effect (2 ADAS-cog score points at 36 months) which might be of the same magnitude as the model misspecification. The performances of IMA in the low typical treatment effect scenarios can be explained by the additional degree of freedom brought by the mixture model, allowing some over-fitting associated with a much lower OFV, misleading the AIC selection process.

Aside from these two specific simulation scenarios, the RMSE was overall better for rcLRT. This loss in accuracy for IMA can be explained by the fact that the formula used to compute the final treatment effect combines two parameters estimates: the treatment effect estimate and the mixture proportion, contrary to rcLRT, where the treatment effect is only in the treatment effect estimate (see Equation (10)).

Overall the performances of the approaches were well aligned with the OFV obtained for each approach with a single fit of the different model (results presented in Appendix D). The usage of real data together with a model that was developed, assessed, and published using the same data frames, this work in an interestingly realistic context with real-life model misspecifications. In contrast, the addition of a simulated treatment effect to create scenarios for power and accuracy assessment might lack some real-life complexity. Nonetheless, it allowed the highlighting of the dangerous combination between described features of the natural history data by the placebo model and greedy behavior of the test statistic (dOFV) and/or selection criteria (AIC).

The scope of this work was restricted to treatment effects for balanced two-armed designs. While it is difficult to extrapolate the results further for most of the approaches, IMA and the standard approach without the selection step were assessed regarding type I error in unbalanced designs with respect to treatment effect and dose-response elsewhere [19]. The results were consistent with the ones presented here.

## 5. Conclusions

This work compared seven NLMEM approaches to test for treatment effects in the same framework using real natural history data. All approaches but IMA and rcLRT had inflated type I error. This can be explained by the misspecification of the placebo model, arising from the use of real natural history data, absent from the previous assessments of cLRT and MAD. Under such circumstances, the five remaining approaches (STDs, SSs, MAD, MAPD, and cLRT) suffered from the greedy behavior of the AIC criteria in the selection or the weighting step, often dismissing the null hypothesis. rcLRT handles the placebo model misspecification by calibrating the cut-off values for the statistical test via a randomization test, while IMA handles it by introducing the drug model already in the null hypothesis via a mixture model. Both IMA and rcLRT show promising results regarding power, bias, and accuracy using natural history data modified by the addition of various simulated treatment effects. However, both approaches were not flawless: IMA had low power to detect low typical treatment effect, and both showed poor performances in the scenarios combining low typical treatment effect and a treatment effect addition similar to the placebo model.

## Figures and Tables

**Table 1:** Alternative disease models for the model averaging across placebo and drug models approach.

Modified Component	Modification
Structural model	Time-exponential
RUV model	IIV on RUV
IIV model	Boxcox transformation of $\eta_{1}$
IIV model	t-distribution of $\eta_{1}$

IIV: Inter-individual variability, RUV: Residual unexplained variability.

**Table 2:** Type I error per approach using the real natural history data (N = 100).

Approach	Placebo Model	Type I Error (%) [1.64–11.28% *]
STDs	Published	100
SSs	Published	17
cLRT	Published	100
rcLRT	Published	6
MAD	Published	100 ^†^
MAPD	Pre-selected set	100 ^†^
IMA	Published	6

* 2.5th and 97.5th percentiles of a binomial distribution with a probability of success of 5% on 100 trial replicates. † Average of the percentage of the relative weights assigned to any H1.

**Table 3:** Power and RMSE for approaches with controlled type I error on data modified by the addition of simulated treatment effect models for the eight investigated scenarios (N = 100). RMSE: root mean squared error, IIV: inter-individual variability.

		rcLRT		IMA	
Simulation Model	Typical Treatment Effect	Power (%)	RMSE	Power (%)	RMSE
Offset	2	100	0.26	37	0.83
Offset IIV	2	100	0.42	33	0.75
Time-linear	2	6	1.29	63	1.54
Time-linear IIV	2	6	1.29	67	1.58
Offset	8	100	0.26	100	0.48
Offset IIV	8	100	0.29	100	0.41
Time-linear	8	100	0.55	100	0.57
Time-linear IIV	8	100	0.57	100	0.61

## Data Availability

Restrictions apply to the availability of these data. Data were obtained from the Alzheimer’s Disease Neuroimaging Initiative (ADNI) and are available on request at https://adni.loni.usc.edu/, accessed on 11 August 2022.

## Abbreviations

    The following abbreviations are used in this manuscript:

ADAS-cog	Alzheimer’s Disease Assessment Scale-cognitive
AIC	Akaike Information Criteria
BMMS	Baseline Mini-Mental State
cLRT	Combined Likelihood Ratio Test
dOFV	Difference in Objective Function Value
IIV	Inter-Individual Variability
IMA	Individual Model Averaging
LRT	Likelihood Ratio Test
MAD	Model Averaging Across Drug models
MAPD	Model Averaging Across Placebo and Drug models
NLMEM	Non-Linear Mixed Effects Models
OFV	Objective Function Value
rcLRT	Randomized Combined Likelihood Ratio Test
RMSE	Root Mean Squared Error
RUV	Residual Unexplained Variability
SSs	structural similarity selection
STDs	Standard model selection

## Appendix B. NONMEM Code of the Published Placebo Model

$PROBLEM    Published model
$INPUT      C ID TIME DV BMMS INVF AGE APOF SEX EDU ARM
;ID   : 817 individuals
;TIME : months
;DV   : ADAS-cog score
;BMMS : baseline MMSE
;INVF : inverse of baseline ADAS
;AGE  : years
;APOF : ApoE 0=non carrier, 1=hetero, 2=homo-carrier
;SEX  : 1=male
;EDU  : education level in years
;ARM  : fake random TRT allocation
$DATA     ../data/data.csv IGNORE=@
$ABBREVIATED COMRES=3 PROTECT
$PRED
; --------- Baseline model
INT=THETA(2)    ;baseline ADAS-cog
BSLP = THETA(3)
MM1 = BSLP*BMMS
BSL = (INT + MM1) + ETA(1)
; --------- Covariates
BAS1=INVF**THETA(5)
;age effect
AGE1 = (AGE/75)**THETA(6)
;ApoE effect
APF = 0
IF(APOF.GT.0)THEN   ;0=non-carrier
APF = 1
ENDIF
APO = THETA(7)
;SEX effect
GEN = 0
IF(SEX.EQ.1)THEN   ;1=male
GEN = 1
ENDIF
GEN1 = THETA(8)**GEN
;education
EDC = (EDU/15)**THETA(9)
; --------- Disease progression model
SLP=THETA(1)/12   ; disease progression
ISLP =SLP*BAS1*AGE1*(APO**APF)*GEN1*EDC+ ETA(2)
ADASCOG=BSL + ISLP*TIME
F=ADASCOG
W=THETA(4)
Y=F+W*EPS(1)
$THETA  4 ; PRM TH1 PLB SLOPE
 (0,60) ; PRM TH2 BASE INTERCEPT
 -1.69 ; PRM TH3 BASE SLOPE
 (0,3) ; PRM TH4 RUV ADD
 (1,3,5) ; PRM TH5 COV GAM INVF
 -1 ; PRM TH6 COV AGE
 1 ; PRM TH7 COV APO
 1 ; PRM TH8 COV SEX
 0 FIX ; PRM TH9 COV EDU
$OMEGA  BLOCK(2)
 9  ; PRM OM1 BASE
 0.01 0.09  ; PRM OM2 PLB SLOPE
$SIGMA  1  FIX  ;   PRM SIG1
$ESTIMATION MAXEVAL=9999 METHOD=1 NOABORT

## Appendix D. Models Description

### Appendix D.1. Models Fitted on Natural History Data

**Table A1:** Models summary of the models fitted to the natural history data for all the approaches but MAPD (n = 1). IIV: inter-individual variability, OFV: objective function value, dOFV: difference in OFV between the model and its reference (Ref), Prm_nb: number of parameters estimated.

Run_nb	Ref	Description	Prm_nb	OFV	dOFV
Models fitted on real natural history data					
2	NA	Published model	11	13,768.66	-
3	2	Published + Offset	12	13,767.64	−1.02
4	2	Published + Offset IIV	13	13,746.54	−22.12
5	2	Published + Disease modifying	12	13,765.02	−3.64
6	2	Published + Disease modifying IIV	13	13,635.12	−133.54
Models fitted on simulated natural history data					
7	NA	Published model on simulated data	11	13,567.91	-
12	7	Published + Offset	12	13,567.91	0
13	7	Published + Offset IIV	13	13,567.91	0
14	7	Published + Disease modifying	12	13,561.34	−6.57
15	7	Published + Disease modifying IIV	13	13,557.64	−10.27
IMA models fitted on real natural history data					
100	NA	Published + Offset base	12	13,765.13	-
101	100	Published + Offset full	13	13,765.11	−0.02
102	100	Published + Offset IIV base	13	13,695.61	−69.52
103	102	Published + Offset IIV full	14	13,694.39	−1.23
104	100	Published + Disease modifying base	12	13,473.09	−292.04
105	104	Published + Disease modifying full	13	13,471.41	−1.67
106	104	Published + Disease modifying IIV base	13	13,455.68	−17.41
107	106	Published + Disease modifying IIV full	14	13,454.20	−1.48
SSs models fitted on real natural history data					
50	NA	Published + Offset base	12	13,766.41	-
54	50	Published + Offset full	13	13,766.40	−0.01
51	50	Published + Offset IIV base	13	13,729.93	−36.48
56	51	Published + Offset IIV full	15	13,728.82	−1.11
52	50	Published + Disease modifying base	12	13,686.89	−79.53
57	52	Published + Disease modifying full	13	13,683.10	−3.79
55	50	Published + Disease modifying IIV base	13	13,182.59	−583.82
58	55	Published + Disease modifying IIV full	15	13,180.55	−2.05

**Table A2:** Models summary of the models fitted to the natural history data for the MAPD approach (n = 1). dOFV: difference in OFV between the model and its reference (Ref), IIV: inter-individual variability, OFV: objective function value, Prm_nb: number of parameters estimated, RUV: residual unexplained variability.

Run_nb	Ref	Description	Prm_nb	OFV	dOFV
Published placebo model					
2	NA	Published placebo model	11	13,768.66	-
3	2	Published + Offset	12	13,767.64	−1.02
4	2	Published + Offset IIV	13	13,746.54	−22.12
5	2	Published + Disease modifying	12	13,765.02	−3.64
6	2	Published + Disease modifying IIV	13	13,635.12	−133.54
Published placebo model with t-distribution transformation					
19	NA	Alternative placebo model	12	13,708.54	-
203	19	Pub t-dist + Offset	13	13,707.82	−0.72
204	19	Pub t-dist + Offset IIV	14	13,691.71	−16.83
207	19	Pub t-dist + Disease modifying	13	13,705.21	−3.33
208	19	Pub t-dist + Disease modifying IIV	14	13,585.26	−123.28
Published placebo with IIV on RUV					
240	NA	Alternative placebo model	12	13,708.51	-
241	240	Pub IIV on RUV + Offset	13	13,708.01	−0.5
242	240	Pub IIV on RUV + Offset IIV	14	13,708.01	−0.5
243	240	Pub IIV on RUV + Disease modifying	13	13,707.30	−1.21
244	240	Pub IIV on RUV + Disease modifying IIV	14	13,639.67	−68.84
Published placebo model with Boxcox transformation					
250	NA	Alternative placebo model	12	13,711.92	-
251	250	Pub Boxcox + Offset	13	13,710.97	−0.94
252	250	Pub Boxcox + Offset IIV	14	13,692.13	−19.78
253	250	Pub Boxcox + Disease modifying	13	13,709.59	−2.32
254	250	Pub Boxcox + Disease modifying IIV	14	13,590.41	−121.51
Published placebo with time-exponential					
270	NA	Alternative placebo model	12	13,745.04	-
271	270	Pub time-exp + Offset	13	13,738.74	−6.3
272	270	Pub time-exp + Offset IIV	14	13,724.31	−20.73
273	270	Pub time-exp + Disease modifying	13	13,737.09	−7.95
274	270	Pub time-exp + Disease modifying IIV	14	13,616.90	−128.14

### Appendix D.2. rcLRT Models Fitted on Data Modified by the Addition of a Simulated Treatment Effect

**Table A3:** Models summary of the rcLRT models fitted to the data modified by the addition of various simulated treatment effects (n = 1). CV: coefficient of variation, dOFV: difference in OFV between the model and its reference (Ref), IIV: inter-individual variability, OFV: objective function value, Prm_nb: number of parameters estimated, RUV: residual unexplained variability, TDE: typical drug effect.

Run_nb	Ref	Description	Prm_nb	OFV	dOFV
Data modified by the addition of offset drug model, TDE = 2					
44	NA	Published placebo model	11	13,844.33	-
170	44	Published plb + Offset	12	13,765.80	−78.53
171	44	Published plb + Offset IIV	13	13,761.33	−83
172	44	Published plb + Time linear	12	13,834.29	−10.04
173	44	Published plb + Time linear IIV	13	13,830.05	−14.28
Data modified by the addition of offset IIV drug model, TDE = 2 with 30%CV					
24	NA	Published placebo model	11	13,858.20	-
175	24	Published plb + Offset	12	13,782.99	−75.21
174	24	Published plb + Offset IIV	13	13,776.00	−82.2
176	24	Published plb + Time linear	12	13,848.41	−9.79
177	24	Published plb + Time linear IIV	13	13,781.10	−77.1
Data modified by the addition of time-linear drug model, TDE = 2					
74	NA	Published placebo model	11	13,768.85	-
179	74	Published plb + Offset	12	13,768.84	−0.01
180	74	Published plb + Offset IIV	13	13,763.69	−5.16
178	74	Published plb + Time linear	12	13,765.69	−3.16
181	74	Published plb + Time linear IIV	13	13,762.61	−6.24
Data modified by the addition of time-linear IIV drug model, TDE = 2 with 30%CV					
64	NA	Published placebo model	11	13,771.71	-
183	64	Published plb + Offset	12	13,771.70	−0.01
184	64	Published plb + Offset IIV	13	13,766.42	−5.3
185	64	Published plb + Time linear	12	13,768.81	−2.91
182	64	Published plb + Time linear IIV	13	13,765.42	−6.29
Data modified by the addition of offset drug model, TDE = 8					
360	NA	Published placebo model	11	15,077.75	-
364	360	Published plb + Offset	12	13,765.80	−1311.95
368	360	Published plb + Offset IIV	13	13,761.33	−1316.41
372	360	Published plb + Time linear	12	14,857.43	−220.32
376	360	Published plb + Time linear IIV	13	14,845.45	−232.3
Data modified by the addition of offset IIV drug model, TDE = 8 with 30%CV					
361	NA	Published placebo model	11	15,185.64	-
365	361	Published plb + Offset	12	13,981.87	−1203.77
369	361	Published plb + Offset IIV	13	13,917.96	−1267.68
373	361	Published plb + Time linear	12	14,987.39	−198.25
377	361	Published plb + Time linear IIV	13	14,961.95	−223.69
Data modified by the addition of time-linear drug model, TDE = 8					
362	NA	Published placebo model	11	13,893.49	-
366	362	Published plb + Offset	12	13,878.02	−15.47
370	362	Published plb + Offset IIV	13	13,873.39	−20.1
374	362	Published plb + Time linear	12	13,765.69	−127.8
378	362	Published plb + Time linear IIV	13	13,762.61	−130.88
Data modified by the addition of time-linear IIV drug model, TDE = 8 with 30%CV					
363	NA	Published placebo model	11	13,916.08	-
367	363	Published plb + Offset	12	13,902.06	−14.02
371	363	Published plb + Offset IIV	13	13,896.40	−19.68
375	363	Published plb + Time linear	12	13,799.26	−116.82
379	363	Published plb + Time linear IIV	13	13,792.27	−123.81

### Appendix D.3. IMA Models Fitted on Data Modified by the Addition of a Simulated Treatment Effect

**Table A4:** Models summary of the IMA models fitted to the data modified by the addition of various simulated treatment effects (n = 1). CV: coefficient of variation, dOFV: difference in OFV between the model and its reference (Ref), IIV: inter-individual variability, OFV: objective function value, Prm_nb: number of parameters estimated, RUV: residual unexplained variability, TDE: typical drug effect.

Run_nb	Ref	Description	Prm_nb	OFV	dOFV
Data modified by the addition of offset drug model, TDE = 2					
312	NA	Published plb + Offset base	12	13,807.81	-
313	312	Published plb + Offset full	13	13,759.81	−48
324	312	Published plb + Offset IIV base	13	13,721.88	−85.93
325	324	Published plb + Offset IIV full	14	13,712.10	−9.78
326	312	Published plb + Time linear base	12	13,843.79	35.98
327	326	Published plb + Time linear full	13	13,834.31	−9.49
328	326	Published plb + Time linear IIV base	13	13,629.18	−214.61
329	328	Published plb + Time linear IIV full	14	13,627.83	−1.35
Data modified by the addition of offset IIV drug model, TDE = 2 with 30%CV					
304	NA	Published plb + Offset base	12	13,822.38	-
305	304	Published plb + Offset full	13	13,776.66	−45.72
302	304	Published plb + Offset IIV base	13	13,732.38	−90
303	304	Published plb + Offset IIV full	14	13,722.67	−99.7
306	302	Published plb + Time linear base	12	13,857.50	125.11
307	306	Published plb + Time linear full	13	13,848.43	−9.07
308	306	Published plb + Time linear IIV base	13	13,641.31	−216.19
309	308	Published plb + Time linear IIV full	14	13,639.74	−1.57
Data modified by the addition of time-linear drug model, TDE = 2					
816	NA	Published plb + Offset base	12	13,766.88	-
817	816	Published plb + Offset full	13	13,766.46	−0.42
818	816	Published plb + Offset IIV base	13	13,690.94	−75.94
819	818	Published plb + Offset IIV full	14	13,689.29	−1.64
812	816	Published plb + Time linear base	12	13,768.85	1.97
813	812	Published plb + Time linear full	13	13,765.70	−3.15
808	812	Published plb + Time linear IIV base	13	13,557.07	−211.77
809	808	Published plb + Time linear IIV full	14	13,556.76	−0.32
Data modified by the addition of time-linear IIV drug model, TDE = 2 with 30%CV					
852	NA	Published plb + Offset base	12	13,769.77	-
853	852	Published plb + Offset full	13	13,769.39	−0.38
854	852	Published plb + Offset IIV base	13	13,693.59	−76.18
855	854	Published plb + Offset IIV full	14	13,691.98	−1.61
850	852	Published plb + Time linear base	12	13,771.71	1.94
851	850	Published plb + Time linear full	13	13,768.82	−2.9
762	850	Published plb + Time linear IIV base	13	13,558.84	−212.87
763	762	Published plb + Time linear IIV full	14	13,558.49	−0.35
Data modified by the addition of offset drug model, TDE = 8					
552	NA	Published plb + Offset base	12	14,303.38	-
553	552	Published plb + Offset full	13	13,710.38	−593.01
554	552	Published plb + Offset IIV base	13	14,283.48	−19.9
555	554	Published plb + Offset IIV full	14	13,700.30	−583.18
556	552	Published plb + Time linear base	12	15,040.48	737.1
557	556	Published plb + Time linear full	13	14,854.14	−186.34
558	556	Published plb + Time linear IIV base	13	14,933.37	−107.11
559	558	Published plb + Time linear IIV full	14	14,776.40	−156.97
Data modified by the addition of offset IIV drug model, TDE = 8 with 30%CV					
524	NA	Published plb + Offset base	12	14,402.80	-
525	524	Published plb + Offset full	13	13,911.77	−491.03
522	524	Published plb + Offset IIV base	13	14,328.56	−74.24
523	522	Published plb + Offset IIV full	14	13,845.67	−482.89
526	522	Published plb + Time linear base	12	15,144.65	816.08
527	526	Published plb + Time linear full	13	14,984.37	−160.27
528	526	Published plb + Time linear IIV base	13	15,027.12	−117.52
529	528	Published plb + Time linear IIV full	14	14,877.69	−149.43
Data modified by the addition of time-linear drug model, TDE = 8					
844	NA	Published plb + Offset base	12	13,893.24	-
845	844	Published plb + Offset full	13	13,875.75	−17.48
846	844	Published plb + Offset IIV base	13	13,818.27	−74.96
847	846	Published plb + Offset IIV full	14	13,816.83	−1.44
842	844	Published plb + Time linear base	12	13,885.17	−8.06
843	842	Published plb + Time linear full	13	13,762.09	−123.08
848	842	Published plb + Time linear IIV base	13	13,783.23	−101.94
849	848	Published plb + Time linear IIV full	14	13,687.60	−95.63
Data modified by the addition of time-linear IIV drug model, TDE = 8 with 30%CV					
784	NA	Published plb + Offset base	12	13,915.84	-
785	784	Published plb + Offset full	13	13,899.91	−15.93
786	784	Published plb + Offset IIV base	13	13,840.96	−74.88
787	786	Published plb + Offset IIV full	14	13,839.97	−0.99
788	784	Published plb + Time linear base	12	13,907.35	−8.49
789	788	Published plb + Time linear full	13	13,795.58	−111.77
782	788	Published plb + Time linear IIV base	13	13,798.92	−108.43
783	782	Published plb + Time linear IIV full	14	13,705.10	−93.82
